# Internal Architecture of a 3D-Printed Ti-6Al-4V Capsule and Preliminary FEM Simulation of Diffusion: CAD-Informed Modeling with Qualitative Micro-CT Characterization

**DOI:** 10.3390/ma19173758

**Published:** 2026-09-03

**Authors:** Katarzyna Kazimierska, Grzegorz Szala, Adam Mazurkiewicz, Mikołaj Wiśniewski, Igor Olszewski

**Affiliations:** 1 Faculty of Mechatronics, Kazimierz Wielki University, Mikołaja Kopernika 1, 85-074 Bydgoszcz, Poland; gszala@ukw.edu.pl (G.S.); igor@olszewski.cloud (I.O.); 2 Faculty of Mechanical Engineering, Bydgoszcz University of Science and Technology, Al. Prof. S. Kaliskiego 7, 85-796 Bydgoszcz, Poland; adam.mazurkiewicz@pbs.edu.pl; 3 Department of Mechanics, University of Córdoba, Cta. Madrid-Cádiz, Km. 396, 14071 Córdoba, Spain; 4 Alufasady LCC, Nakielska 247, 85-391 Bydgoszcz, Poland; mwisniewski@alufasady.pl

**Keywords:** Ti-6Al-4V, additive manufacturing, direct metal laser sintering, finite-element modeling, diffusion, reservoir–channel architecture, micro-computed tomography, computer-aided design, implantable drug-delivery platform

## Abstract

A central challenge in implantable local-delivery systems is to determine how internal device architecture shapes solute transport before drug-specific loading, release, and biological validation are available. This study examines the reservoir–channel architecture of the previously characterized KTD/KTM Ti-6Al-4V capsule platform and evaluates its effect on diffusion-dominated concentration redistribution in a preliminary finite-element model. Micro-computed tomography (micro-CT) was used for qualitative characterization of the as-fabricated internal architecture, whereas the representative computational geometry was defined from the capsule computer-aided design (CAD) concept and previously reported dimensions. Transport of a nominal, fully dissolved species was modeled in COMSOL Multiphysics 5.2 using Fickian diffusion with a prescribed effective diffusion coefficient (*D_eff_* = 1 × 10^−9^ m^2^/s) and a nominal peak initial concentration scale of 1.0 mol/m^3^. Under the closed no-flux condition, the internal control-point concentration decreased to near zero within approximately 4 h, while the remote outer control point reached approximately 0.002–0.003 mol/m^3^ by 6 h. A one-sided Dirichlet sink produced a more directional concentration field, demonstrating the sensitivity of the simulated distribution to geometry and boundary conditions. These results describe an idealized, CAD-informed diffusion problem and do not represent experimentally validated drug-release kinetics, therapeutic concentrations, or biological performance. The model is therefore intended as a design-phase framework for guiding subsequent drug-specific transport and experimental validation studies.

## 1. Introduction

Local drug delivery is intended to increase drug exposure within a defined tissue region while limiting systemic exposure. For implantable reservoir systems, a key design question is how the internal reservoir, channels, and outlet arrangement shape transport into adjacent tissue before a specific drug formulation and therapeutic dosing regimen are selected [1,2].

Local therapy is particularly relevant when the aim is to increase drug exposure within a confined tissue volume while reducing systemic burden, for example, when residual microscopic disease or limited tissue penetration may compromise local disease control. Reviews of local cancer drug delivery emphasize that implantable depots are intended to maintain drug availability at the site of disease, but that their effectiveness depends on transport, penetration depth, and the spatial range over which therapeutically relevant concentrations can be sustained [1].

Additively manufactured titanium structures combine mechanical robustness with high geometric design freedom, and their pore geometry, surface state, and microstructure can strongly affect mechanical and biological performance [3,4,5,6,7]. In drug-delivery-oriented titanium implants, local release is more commonly enabled by dedicated drug-loaded interfaces, coatings, nanotube-based systems, responsive layers, or reservoir-like designs rather than by titanium porosity alone [8,9,10,11]. Ti-6Al-4V is a well-established material for long-term implantable devices, combining low density and high mechanical strength with chemical stability under physiological conditions [12,13,14,15]. In the present concept, the alloy serves as a durable structural carrier; consequently, its persistence in the body is an accepted characteristic of the device rather than an intended feature of a resorbable delivery system.

From a design standpoint, implantable drug-delivery systems are not governed by material chemistry alone. Contemporary reviews distinguish matrix-type and reservoir-type devices and emphasize that release behavior is shaped by drug properties, material permeability, and device geometry. In three-dimensional printed implants, architecture-specific variables such as internal compartments, implant shape, surface-area-to-volume ratio, and directional diffusion pathways can therefore be used to modulate local drug transport [2,16,17,18].

Within titanium-based implant research, local drug delivery has most often been developed by coupling the structural role of Ti alloys with surface- or near-surface release strategies, including nanotube architectures, coatings, immobilized carriers, and responsive drug-loaded interfaces [8,9]. In anticancer applications, Maher et al. used anodized micro/nanostructured 3D-printed Ti implants loaded with doxorubicin (DOX) or Apo2L/TRAIL [10], Fan et al. combined a 3D-printed titanium scaffold with pH-responsive PEGylated paclitaxel prodrugs [11], and Xiao et al. developed a polydopamine (PDA)-modified Ti-based system for pH-dependent DOX release [19]. The capsule studied here follows a complementary design strategy in which a central reservoir and predefined channel network are treated as structural variables shaping a simulated local concentration field.

Related work has also integrated drug reservoirs directly into additively manufactured titanium implants. Chang et al. reported a patient-specific mandibular Ti implant containing an integrated refillable cisplatin tank, in which local drug release was coupled to reconstructive implant function [20]. The present capsule is conceptually different: it is a standalone perforated Ti-6Al-4V carrier whose internal architecture is qualitatively characterized by micro-CT and represented by a simplified CAD-informed diffusion model.

The external geometry, surface roughness, and compressive mechanical behavior of the direct metal laser sintering (DMLS)-printed Ti-6Al-4V capsule platform were characterized previously [21]. Those data are cited here only as design context; no new mechanical testing or surface-roughness measurements were performed in the present study. The current work focuses on the internal reservoir–channel architecture and its representation in a preliminary diffusion model.

The purpose of the computational analysis is not to establish drug-release kinetics but to examine how a representative reservoir–channel geometry redistributes an initially prescribed concentration field under simplified diffusion-only conditions. Finite-element modeling (FEM) in COMSOL Multiphysics 5.2 (v. 5.2; COMSOL AB, Stockholm, Sweden) can be useful at this design stage, provided that the resulting concentration fields are not interpreted as drug-specific or physiologically validated predictions [22,23].

The intended use case is a small implantable local depot positioned in a surgically accessible post-resection tissue bed, where short-range redistribution into adjacent tissue may be relevant. To avoid implying a clinically validated indication, the present model does not assign a specific cancer type, anatomical site, active pharmaceutical ingredient (API), dose, or therapeutic concentration range because drug-specific transport, tissue uptake, clearance, and biological calibration were not performed. Doxorubicin is used later only to illustrate conversion of the nominal concentration scale between molar and mass units. It is not treated as the calibrated modeled drug and is not compared with a biological efficacy threshold. Accordingly, this study uses the previously characterized KTD/KTM Ti-6Al-4V capsule platform [21] as the structural basis for a CAD-informed, geometry-focused FEM analysis of diffusion-dominated redistribution under idealized boundary conditions.

## 2. Materials and Methods

### 2.1. Capsule Design and Fabrication

The structural platform used in the present modeling study was the previously characterized KTD/KTM Ti-6Al-4V capsule series manufactured by direct metal laser sintering (DMLS) on an Orlas Creator printer (O.R. Lasertechnologie GmbH, Dieburg, Germany) with a layer thickness of 25 µm, laser power of 82 W, and scanning speed of 500 mm/s, using gas-atomized Ti-6Al-4V ELI (Grade 23) feedstock (MetcoAdd^TM^ Ti64 G23-A; Oerlikon Metco, Winterthur, Switzerland) [21]. KTD and KTM are the design identifiers used in the preceding study for the larger and smaller capsule variants, respectively. Their external dimensions, roughness, and compressive performance were reported previously and are used here only as structural context. The larger KTD capsule measured 6.2 mm × Ø11.2 mm and the smaller KTM capsule 4.5 mm × Ø8 mm. The FEM analysis used a simplified representative reservoir–channel geometry rather than a dimensionally exact replica of either variant.

### 2.2. Micro-CT Imaging and Structural Analysis

Micro-computed tomography (micro-CT) data from the previously characterized KTD/KTM capsule platform [21] were used for qualitative assessment of the internal reservoir, channel network, and surface openings. Scanning was performed with a SkyScan 1270 system (Bruker, Billerica, MA, USA) at 80 kV and 50 µA, with voxel sizes of 7.50–8.00 µm. Reconstruction and visualization used NRecon (v. 1.7.4.2), CTan (v. 1.18.8.0+), and CTvox (v. 3.3.0r1403). In the present study, micro-CT was not used to extract quantitative transport descriptors or to generate the FEM geometry directly; rather, it qualitatively confirmed the reservoir–channel architecture defined from the CAD and previously reported dimensions. Quantitative micro-CT descriptors such as pore connectivity, channel-volume distribution, specific surface area, and tortuosity were not determined here [24,25].

### 2.3. Mathematical Model

The computational model was intentionally restricted to diffusion-driven redistribution in order to isolate the effect of geometry. The equations below distinguish the active diffusion model from broader transport formulations that are retained only as theoretical context for future drug- and tissue-specific extensions.

To describe the simulated redistribution of drug from the capsule into the surrounding tissue domain, transport was treated as diffusion-dominated. The model starts from Fick’s second law in its three-dimensional, time-dependent form:

(1) $$\frac{\partial C}{\partial t}=\nabla \cdot (D_{eff}\nabla C)$$

where *C* (mol/m^3^) is the local drug concentration, *t* (s) is time, and *D_eff_* (m^2^/s) is the effective diffusion coefficient. For a porous medium, *D_eff_* is related to the free-solution diffusivity *D* by:

(2) $$D_{eff}=\frac{\varepsilon }{\tau }\cdot D$$

in which ε is the porosity and τ ≥ 1 is the tortuosity factor. Mass exchange between the solid drug phase and the pore fluid can be represented for the solid phase by a first-order depletion expression:

(3) $$\frac{\partial C_{A}}{\partial t}=-k(C_{A}-C_{B})$$

where *C_A_* is drug concentration in the solid carrier phase, *C_B_* is concentration in the pore fluid, and *k* (s^−1^) is the mass-transfer rate constant. The complete mass balance in the pore fluid is:

(4) $$\varepsilon \frac{\partial C_{B}}{\partial t}+u\cdot \nabla C_{B}=\nabla \cdot \left(D_{eff}\nabla C_{B}\right)-\rho_{p}\frac{\partial C_{A}}{\partial t}-R_{B}$$

Equation (4) shows a broader pore-fluid balance that can include advection and biochemical loss, whereas Equation (3) represents a possible solid-phase depletion term. These terms were not activated in the simulations reported here. The implemented model used u = 0 and *R_B_* = 0 and therefore reduced to the pure diffusion form of Equation (1), with *D_eff_* prescribed directly. Drug size, solubility, binding, degradation, tissue uptake, vascular or lymphatic clearance, and interstitial convection were not represented [22,26,27].

Standard lumped release equations commonly used to describe experimental release profiles are also summarized below.

These equations provide reference formulations for zero-order, first-order, and Korsmeyer–Peppas-type release descriptions [28].

The zero-order model expresses the released mass as a linear function of time:

(5) $$M_{t}=k_{0}\cdot t$$

The first-order model describes fractional release as an exponential approach to completion:

(6) $$\frac{M_{t}}{M_{\infty }}=1-exp(-k_{1}\cdot t)$$

The Korsmeyer–Peppas power-law model can be used as a phenomenological description of diffusion- or erosion-influenced release:

(7) $$\frac{M_{t}}{M_{\infty }}=k\cdot t^{n}$$

The zero-order, first-order, and Korsmeyer–Peppas relations are included only as reference formulations for future experimental release studies [28]. No experimental cumulative-release profile was available in the present work, and none of these lumped kinetic models was fitted to the FEM output.

### 2.4. COMSOL Simulation Setup

Numerical simulations were performed in COMSOL Multiphysics 5.2 using the Transport of Diluted Species (TDS) physics interface [29]. Two domains were defined: (i) the capsule interior containing the representative reservoir–channel geometry and (ii) the surrounding tissue-like computational volume. The analysis tracks redistribution of a nominal concentration field between these domains; it does not represent experimentally measured drug loading or tissue concentration. Table 1 summarizes the implemented settings.

In Table 1, *D_eff_* is the effective diffusion coefficient prescribed directly in the active model. The listed porosity (ε) and tortuosity (τ) values are nominal context for the theoretical porous-medium relation and were not used to calculate *D_eff_* in the reported simulations. The concentration entries define the nominal scale of the initialized field, and the boundary conditions define a closed reference case and an idealized one-sided sink.

**Figure 1:**
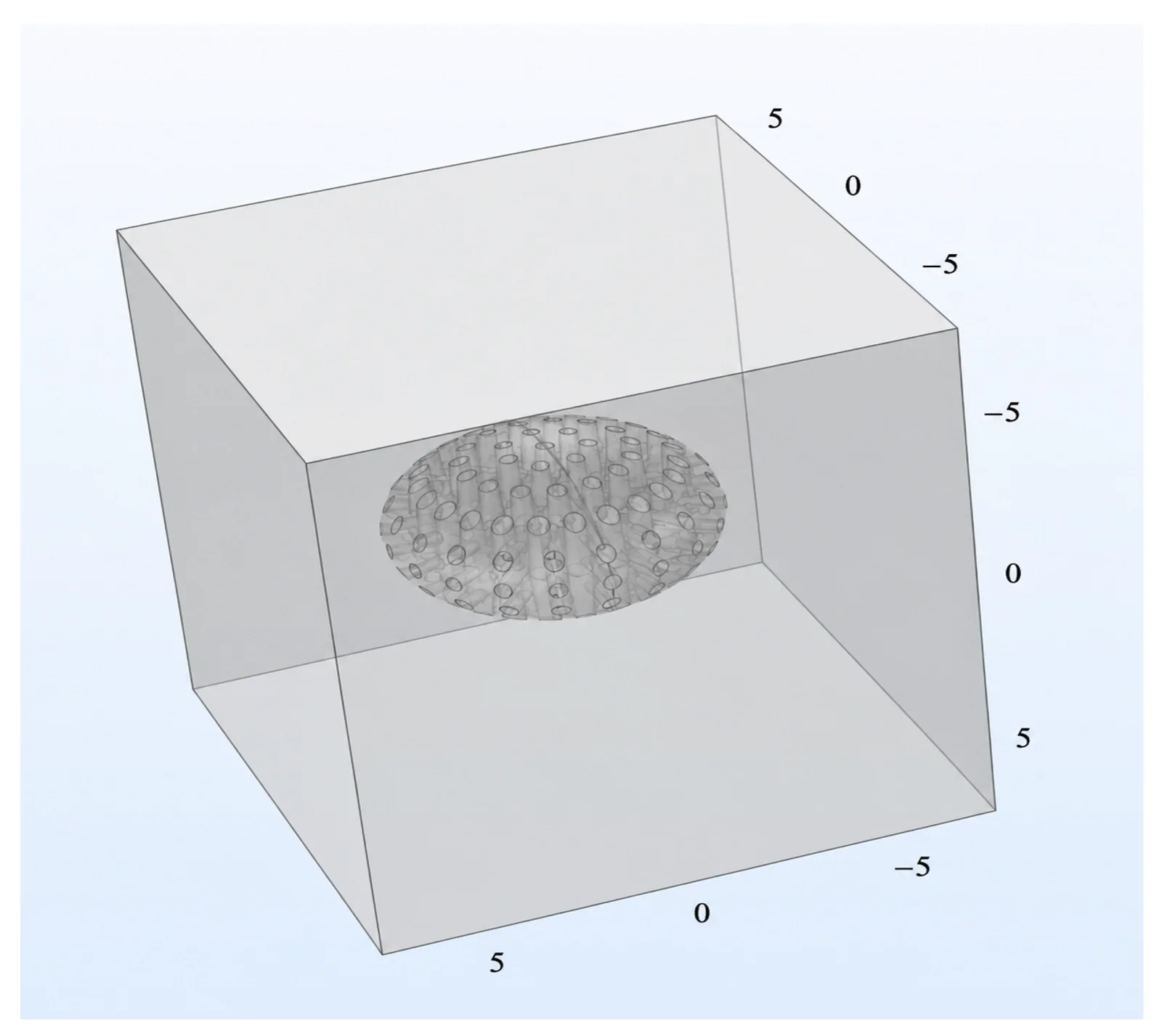
COMSOL Multiphysics 5.2 three-dimensional simulation domain showing the CAD-informed representative Ti-6Al-4V capsule geometry within the surrounding computational domain. The CAD reference geometry had an external envelope of 7.99 mm in diameter and 4.39 mm in height; the displayed tissue-like domain extends approximately ±5 mm along the axes.

The representative three-dimensional capsule geometry (Figure 1) was defined from the existing capsule CAD and dimensions reported in [21], and was then implemented in COMSOL 5.2 as the reservoir–channel geometry used for the preliminary diffusion analysis. Measurements of the supplied CAD representation gave an external envelope of 7.99 mm in diameter and 4.39 mm in height. This closely matches the external scale of the KTM variant (8.0 mm × 4.5 mm; approximately 0.1% difference in diameter and 2.4% in height) and is approximately 29% smaller than the KTD variant in both dimensions. Transverse sections of the CAD geometry showed 23 internal channel cross-sections with diameters of 0.42–0.49 mm. The central void had an effective transverse diameter of approximately 4.0 mm and a chambered, rather than purely cylindrical, form. The outlet-spacing range of 1.0–1.3 mm was retained from the capsule design [21]. Surface micro-roughness, partially fused powder features, and manufacturing tolerances were not represented. Micro-CT was used only for qualitative confirmation of the reservoir–channel architecture. The representative capsule was embedded in a surrounding computational domain extending approximately ±5 mm along each displayed axis.

### 2.5. Boundary and Initial Conditions

The model did not assume a saturated solution or a dissolution-limited solid phase. Instead, c_0_ = 1.0 mol/m^3^ was used as a nominal concentration scale for a fully dissolved species. A discontinuous step assignment of 1.0 mol/m^3^ inside the reservoir and 0 mol/m^3^ outside produced numerical instability at t = 0; therefore, the initial field was regularized with a smooth exponential transition:

(8) $$c\left(x,y,z,0\right)=\exp\left[-385\cdot \sqrt{{\left(x-x_{0}\right)}^{2}+{\left(y-y_{0}\right)}^{2}+{\left(z-z_{0}\right)}^{2}}\right]\;(\mathrm{mol}/m^{3})$$

where (x_0_, y_0_, z_0_) is the geometric center of the capsule. The coefficient −385 defines the steepness of the numerical transition in relation to the coordinate units used in COMSOL 5.2. The value c_0_ = 1.0 mol/m^3^ is therefore the peak nominal concentration scale of the smoothed initial field, not an experimentally measured or spatially uniform drug loading. The smoothing function is an initialization device and does not act as a source term during the simulation. Accordingly, the total initial amount represented by the model is the volume integral of the prescribed initial concentration field, rather than c_0_ multiplied by a physically loaded reservoir volume. In the closed no-flux case, with no active source or sink terms, the integrated solute amount represented by that initialized field is conserved after t = 0; the decline at the central control point reflects redistribution of the numerical field rather than loss of a measured drug dose. Under the one-sided Dirichlet condition (c = 0), solute can leave the computational domain through the selected sink-facing boundary. Both no-flux and Dirichlet cases are idealized mathematical limits and do not represent physiological tissue boundaries, interstitial flow, binding, vascular clearance, or heterogeneous tissue properties [22,26].

### 2.6. Mesh Sensitivity Analysis

**Figure 2:**
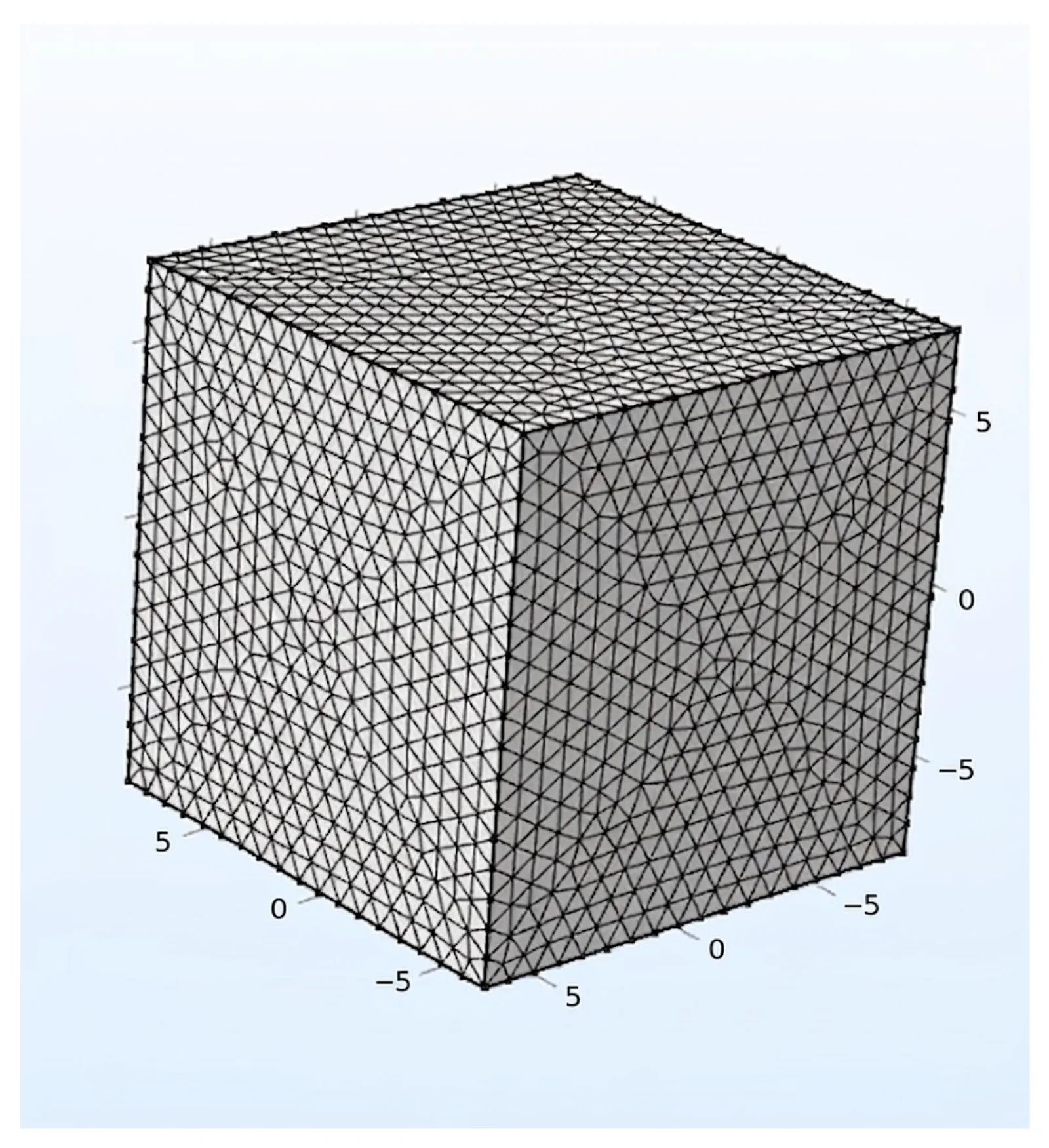
Free-tetrahedral mesh (Finer setting) applied to the 3D simulation domain. The refined mesh density was used to resolve steep concentration gradients at the capsule-tissue interfaces.

Mesh sensitivity was assessed using three physics-controlled free-tetrahedral settings: Extremely Coarse, Normal, and Finer. The Extremely Coarse mesh produced COMSOL 5.2 warnings associated with narrow channel features, including edges or faces smaller than the specified minimum element size. Normal and Finer meshes were compared using the concentration–time profiles at the internal and remote control points. The comparison used the practical decay time of the internal Point 724 and the 6 h concentration at the remote Point 1369, providing a quantitative solution-level check of the effect of refinement on the retained COMSOL 5.2 point-graph outputs. The Finer setting eliminated the reported coarse-mesh warnings and produced the smoothest profiles, and was therefore used for the reported simulations. The Finer mesh is shown in Figure 2.

## 3. Results

### 3.1. Capsule Architecture Used for FEM Model Definition

**Figure 3:**
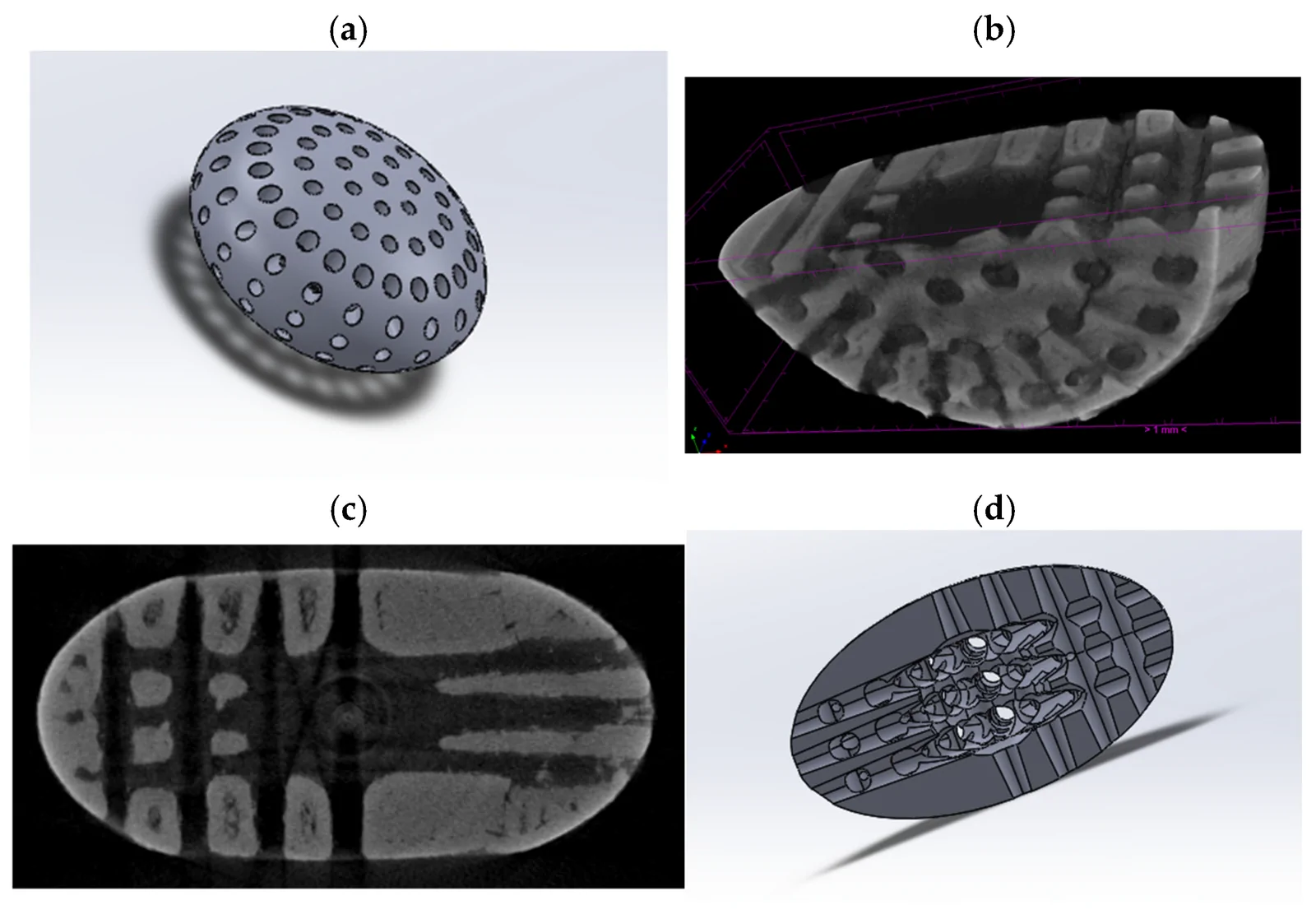
Structural basis of the previously characterized Ti-6Al-4V capsule platform: (**a**) as-designed CAD isometric view of the external capsule geometry; (**b**) three-dimensional micro-CT reconstruction of the as-fabricated capsule showing the internal reservoir–channel architecture; (**c**) orthographic micro-CT section of the as-fabricated capsule; and (**d**) CAD cross-section showing the central reservoir, radial–axial channels, and surface openings. The micro-CT panels provide qualitative structural characterization and were not used to derive quantitative FEM dimensions.

This section summarizes the structural features relevant to the FEM representation. External dimensions, roughness, and mechanical performance were reported previously for the KTD/KTM capsule series [21]; no new mechanical or roughness measurements were performed here. Complete external views of the as-fabricated KTD/KTM capsules were documented in the preceding experimental characterization [21]. Figure 3 distinguishes the as-designed CAD structure from the qualitative micro-CT views of the as-fabricated internal architecture, while Table 2 summarizes the previously reported KTD/KTM geometric parameters used as design context. The present analysis focuses on the reservoir, channel network, and surface openings as the structural basis of the simplified diffusion pathway.

The macro-scale architecture consists of a central reservoir connected to the external surface by a three-dimensional radial–axial channel network and multiple openings. In a future drug-loaded implementation, the reservoir would necessarily be filled after DMLS fabrication through accessible openings because the metal-printing process is incompatible with pre-loading a drug. No loading procedure, loading efficiency, retention measurement, or release experiment was performed in the present study; the computational initial condition is therefore a nominal concentration field rather than a measured loading state.

At the micro-scale, the channel walls show irregular powder-like features consistent with the DMLS surface morphology previously documented for this capsule series; their identity and quantitative contribution to transport were not determined here. The previously reported roughness value (Rz = 118.9 µm) was measured on a same-process flat reference specimen because capsule curvature limited direct profilometry [21]. These observations provide qualitative design context only. Surface opening diameters and spacing reported previously were Ø0.50 mm and 1.3 mm for KTD and Ø0.40 mm and 1.0 mm for KTM [21].

### 3.2. Concentration Distribution Under No-Flux Conditions

**Figure 4:**
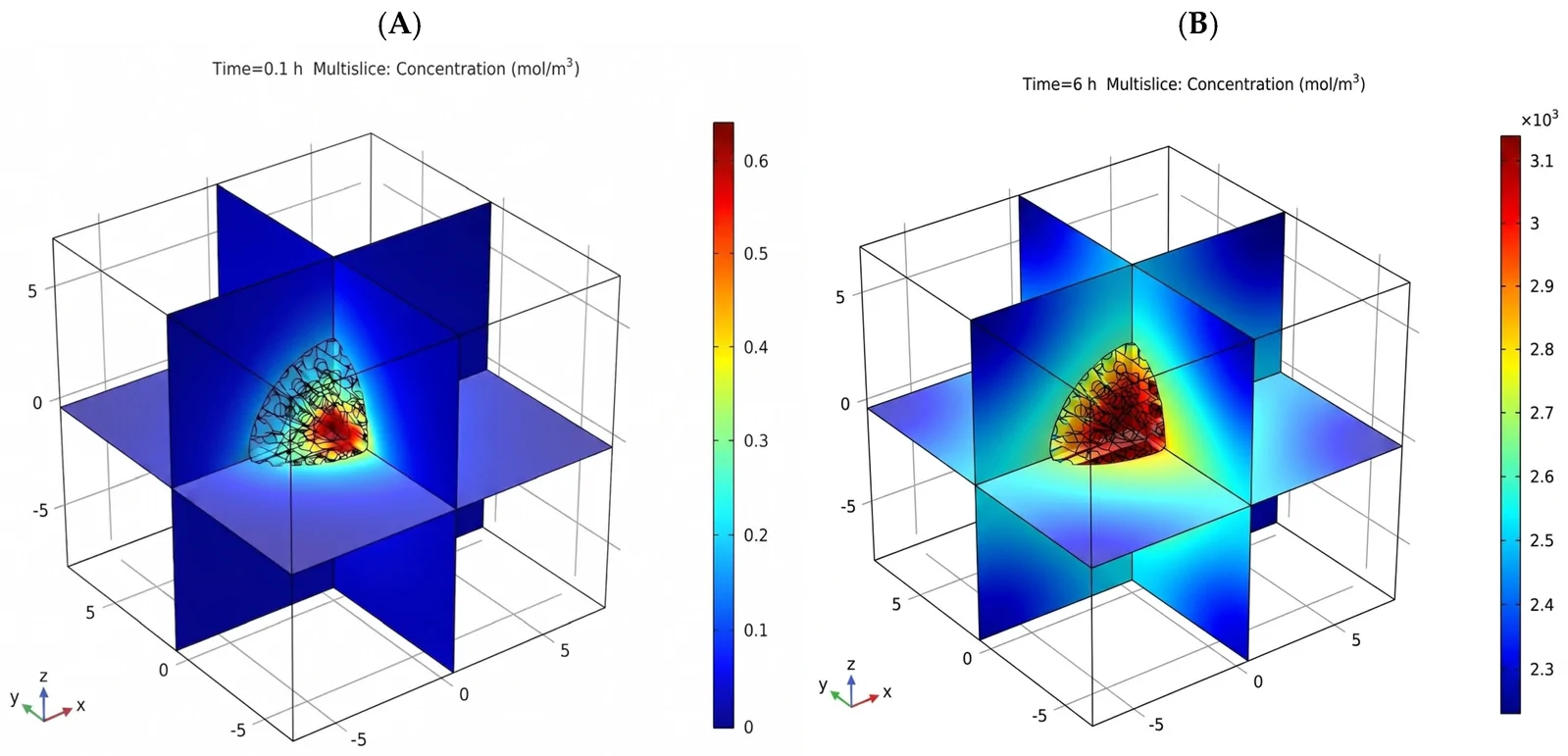
COMSOL Multiphysics 5.2 simulation of the modeled concentration field (mol/m^3^) under the no-flux outer boundary condition; (**A**) t = 0.1 h, (**B**) t = 6 h. The non-uniform outlet arrangement is reflected in the spatial concentration gradients.

Figure 4 shows the spatial distribution of the modeled concentration field at t = 0.1 h and t = 6 h under no-flux outer boundary conditions.

At t = 0.1 h, the highest modeled concentration remains near the capsule interior, with steep gradients at the channel openings. By t = 6 h, the central concentration has decreased and the concentration field has spread through the surrounding domain. The distribution reflects the non-uniform arrangement of outlets, producing spatially non-uniform gradients in the idealized diffusion model.

### 3.3. Open-Wall Directional Diffusion Case

**Figure 5:**
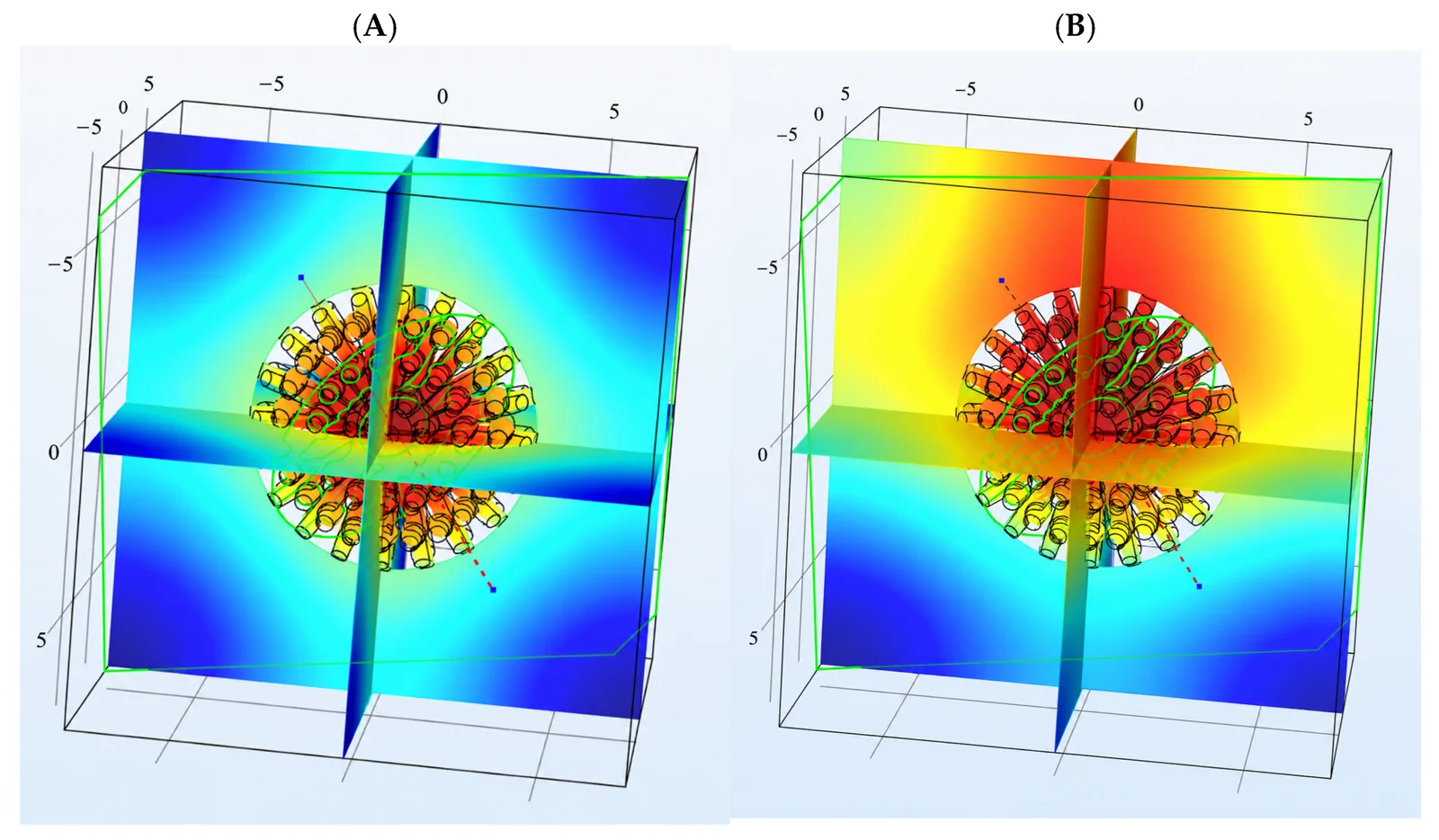
Idealized one-sided sink simulation; (**A**) t = 6 h, (**B**) t = 12 h. The Dirichlet condition (c = 0) on the selected outer face produces an increasingly directional concentration gradient toward the sink-facing boundary.

**Figure 6:**
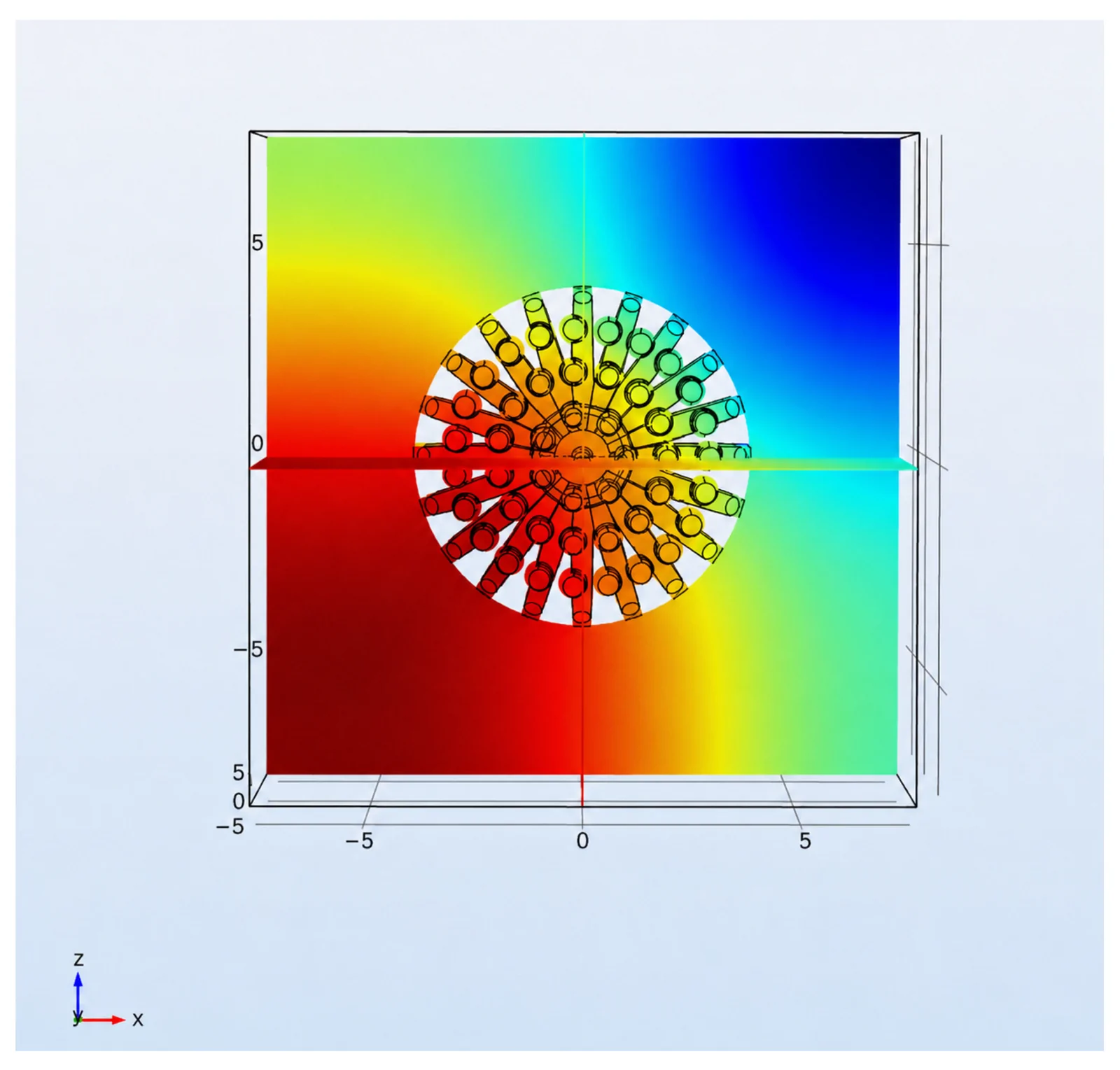
Planar (z–x) view of the modeled concentration field at t = 12 h with the one-sided Dirichlet boundary condition. The asymmetric external sink produces a directional gradient; the internal radial channel arrangement remains visible in cross-section.

Figure 5 and Figure 6 show the idealized one-sided Dirichlet condition, in which c = 0 was imposed on one external face. At t = 6 h, the concentration field begins to bias toward the sink-facing side; by t = 12 h, the asymmetry is more pronounced. This case demonstrates sensitivity of the simulated field to an imposed asymmetric boundary and should not be interpreted as a physiological clearance model.

**Figure 7:**
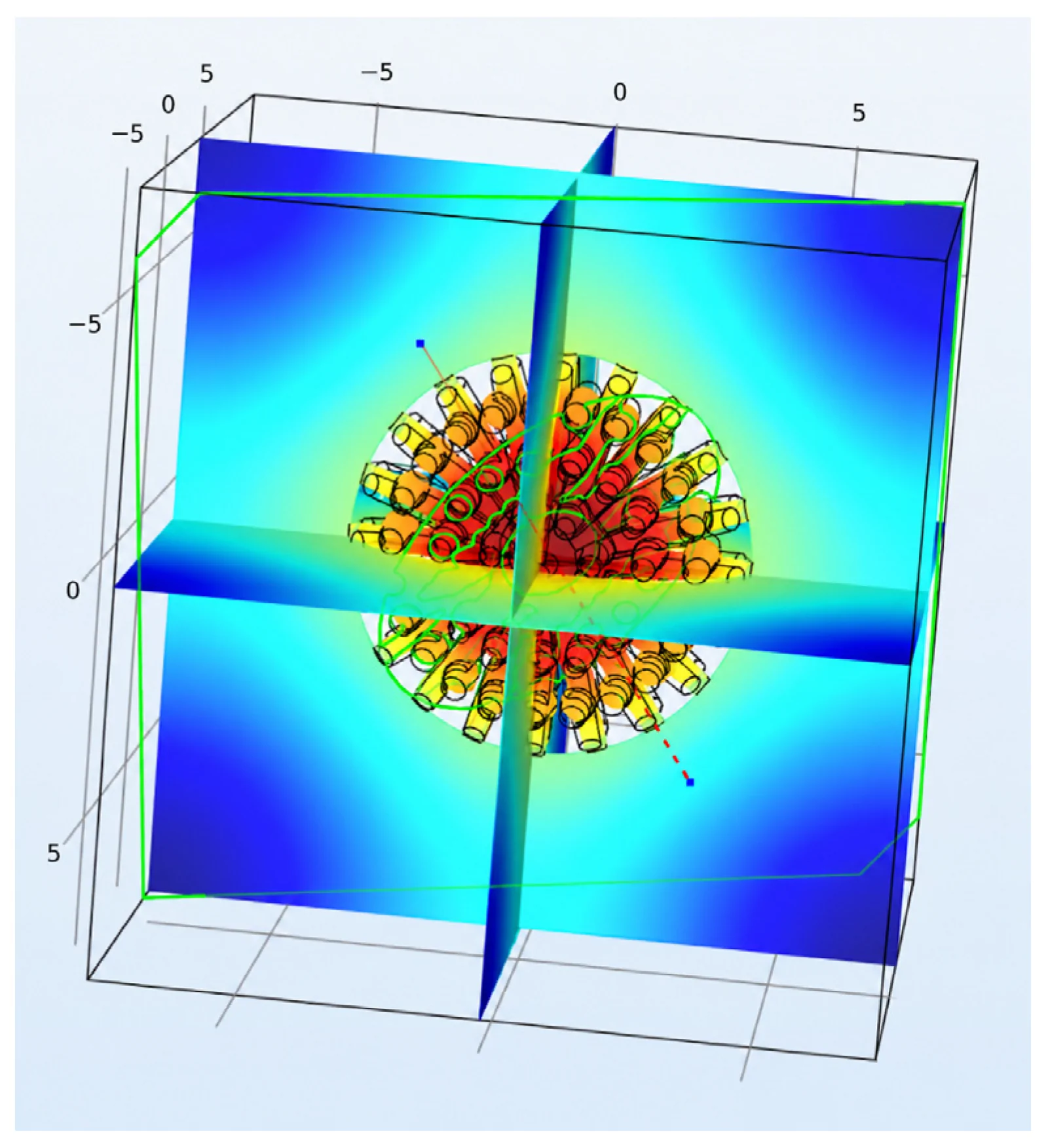
Reference no-flux simulation at t = 10 h. The concentration field remains less directionally biased than in the one-sided Dirichlet case shown in Figure 5.

The reference case without an open wall at t = 10 h is shown in Figure 7. Comparison with the open-wall simulations illustrates that the imposed Dirichlet sink increases transport towards that side of the domain and locally steepens the concentration gradient.

### 3.4. Concentration–Time Profiles

**Figure 8:**
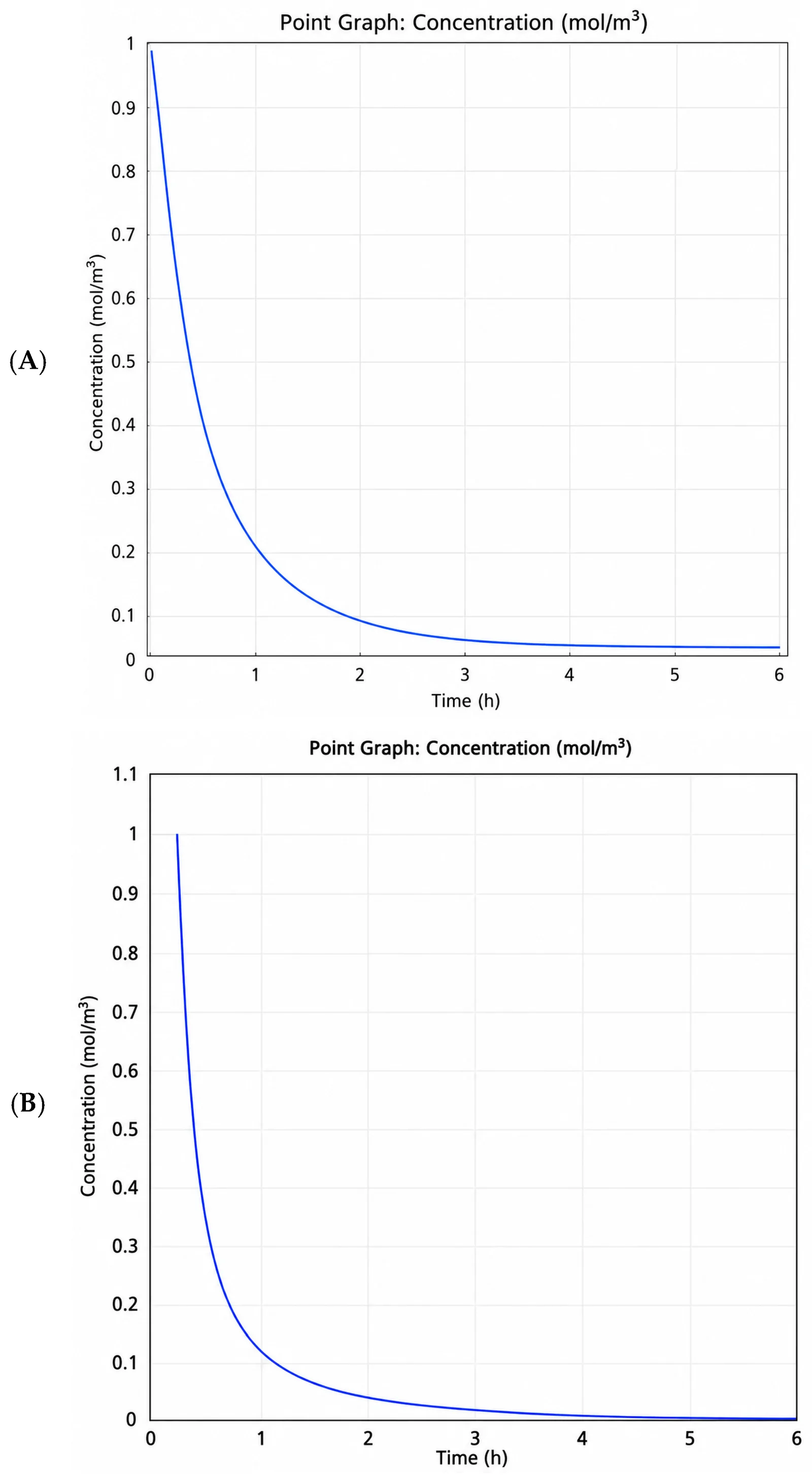
Modeled concentration versus time at the internal computational control point (Point 724). (**A**) Normal mesh. (**B**) Finer mesh. The local concentration decreases to near zero within approximately 4 h for the selected nominal parameter set.

Concentration–time profiles were extracted at two computational control points selected to represent contrasting locations rather than clinically calibrated sampling sites. Point 724, near the capsule center, monitors redistribution away from the initially highest-concentration region; its value decreases from the nominal peak scale to near zero within approximately 4 h (Figure 8).

**Figure 9:**
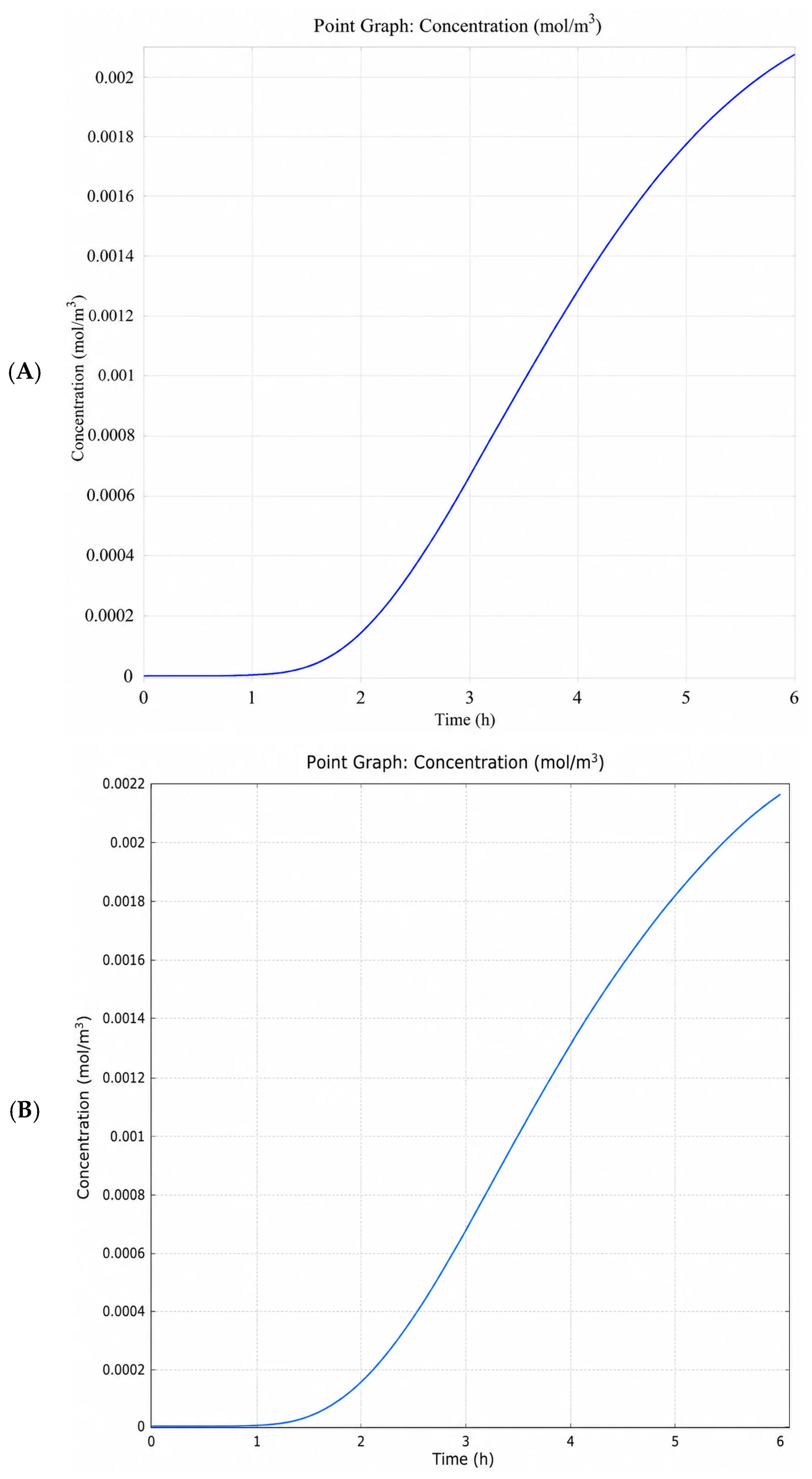
Modeled concentration versus time at the remote outer control point (Point 1369). (**A**) Normal mesh. (**B**) Finer mesh. The concentration reaches approximately 0.002–0.003 mol/m^3^ by 6 h for the selected nominal parameter set. This point is a computational probe and not a calibrated tissue concentration.

Point 1369 is located at a remote corner of the surrounding computational domain and was selected as a deliberately distant monitoring location. It does not represent a clinically calibrated tissue concentration, and its late-time value is sensitive to the finite domain and boundary conditions. The modeled concentration rises from approximately zero to 0.002–0.003 mol/m^3^ (2–3 µM) by approximately 5–6 h; a fully established plateau is not demonstrated in the available time window (Figure 9).

### 3.5. Mesh Sensitivity Results

The Extremely Coarse mesh produced numerical warnings at narrow geometric features and was considered unsuitable. The Normal and Finer meshes produced closely matching control-point outputs. At the remote Point 1369, the 6 h concentration was approximately 0.002 mol/m^3^ for both meshes, with no difference distinguishable at the resolution of the retained point-graph outputs (grid resolution ≈ 0.0002 mol/m^3^). At the internal Point 724, both meshes gave the same practical time scale for concentration to decrease to near zero, approximately 4 h, with no material shift. These comparisons, summarized in Table 3, indicate that the representative outputs are effectively intensive to refinement from Normal to Finer at the level resolved by the retained results. The Finer free-tetrahedral setting was retained for the reported simulations because it also eliminated the coarse-mesh warnings and produced the smoothest profiles.

## 4. Discussion

The structural observations support representing the device as a reservoir–channel system for design-phase transport analysis. The previously reported opening diameters (Ø0.40–0.50 mm) and spacing define discrete pathways through the metallic shell, but the present simulations do not establish an experimental release rate or a rate-controlling mechanism. The roughness value reported previously [21] is retained only as material and design context. More broadly, studies of implantable delivery systems, including 3D-printed and ceramic devices, show that geometry, exposed surface area, microstructure, and transport-path architecture can influence local transport and release behavior [16,18,20,30,31].

Within the assumptions of the model, Fickian diffusion with a prescribed *D_eff_* provides a simple way to examine how a representative reservoir–channel geometry redistributes an initial concentration field. The principal numerical observation is that the non-uniform arrangement of channels and openings produces spatially non-uniform gradients, while an asymmetric external sink further biases the field. These results support using geometry as a design variable in subsequent, better calibrated models; they do not demonstrate controlled release in the pharmaceutical sense.

The no-flux and one-sided Dirichlet conditions should be interpreted as mathematical limiting cases. The former prevents external mass loss, whereas the latter imposes zero concentration on one selected face. Neither represents the combined effects of interstitial flow, extracellular-matrix resistance, binding, vascular or lymphatic clearance, and heterogeneous tissue properties. Consequently, the directional fields shown here are useful for sensitivity analysis but cannot be translated directly to an in vivo concentration distribution [22,26,27].

The concentration–time profiles provide numerical indicators for the selected nominal parameter set: the internal control point approaches zero within approximately 4 h, while the remote point reaches 0.002–0.003 mol/m^3^ (2–3 µM) by approximately 6 h. These values are not universal release times or therapeutic concentrations. For scale only, if the nominal species is interpreted as doxorubicin free base (molar mass 543.52 g/mol), c_0_ = 1.0 mol/m^3^ corresponds to approximately 0.544 mg/mL, and 0.002–0.003 mol/m^3^ corresponds to approximately 1.09–1.63 µg/mL. These conversions are provided solely to indicate the numerical scale of the modeled field in mass and molar units. They are not intended as a comparison against a biological efficacy threshold and do not imply therapeutic activity. Because the active diffusion model is linear in concentration, the modeled field scales linearly with the prescribed initial amplitude c_0_ under otherwise unchanged parameters. A clinically required c_0_ cannot be inferred without drug- and tissue-specific calibration.

Experimental titanium-based delivery systems provide useful qualitative context but are not directly comparable to the present calculation because they involve specific drugs, coatings, formulations, and cumulative-release measurements [10,11,19,20]. For example, nanotube-functionalized titanium systems have shown multistage release extending beyond the first hours [10], whereas the current model describes short-term redistribution of a prescribed concentration field in a simplified reservoir–channel geometry. The comparison therefore supports only the broader design concept and not equivalence of release kinetics. Reservoir-type polymeric implants with rate-controlling porous membranes and externally triggered nanofiber/hydrogel platforms illustrate other mechanistically distinct routes to localized or sustained delivery [32,33]; they provide design context but do not validate the present diffusion model.

The main limitations are the prescribed homogeneous isotropic *D_eff_*, the smoothed nominal initial field, omission of drug-specific dissolution, binding, degradation, uptake and clearance, absence of interstitial convection, qualitative rather than quantitative use of micro-CT, finite surrounding-domain size, and absence of experimental loading or release validation. No cytotoxicity, biocompatibility, tumor-cell response, or tissue-interaction experiments were performed in this study. The characteristic diffusion length scale after 6 h, √(*D_eff_* t) ≈ 4.6 mm, is comparable to the approximately 5 mm half-domain extent; therefore, the late-time concentration at the remote corner should be regarded as domain-sensitive, and invariance to a larger tissue domain was not demonstrated. Future work should calibrate transport parameters for a defined API, quantify the actual capsule geometry from micro-CT or CAD/COMSOL data, and validate concentration–time behavior experimentally [22,24,25,27,34,35].

## 5. Conclusions

This preliminary study combines qualitative micro-CT characterization with CAD-informed finite-element modeling to examine diffusion through the reservoir–channel architecture of a 3D-printed Ti-6Al-4V capsule. The principal conclusions are:

Qualitative micro-CT confirms the central reservoir, radial–axial channel network, and surface openings of the previously characterized capsule platform, whereas the computational geometry is CAD-informed rather than quantitatively derived from micro-CT.

External dimensions, roughness, and mechanical properties cited in this work were reported previously [21] and are included only as design context; no new mechanical or roughness measurements were performed.

For the selected nominal parameter set (*D_eff_* = 1 × 10^−9^ m^2^/s), the internal computational control point decreases to near zero within approximately 4 h, while the remote outer point reaches approximately 0.002–0.003 mol/m^3^ by approximately 6 h. These are model outputs, not experimentally validated release kinetics.

The non-uniform outlet arrangement produces spatially non-uniform concentration gradients, and the one-sided Dirichlet sink further increases directional bias in the simulated field.

The no-flux and Dirichlet conditions are idealized computational limits; physiological transport, clearance, binding, tissue heterogeneity, and drug-specific properties were not represented.

Normal-to-Finer refinement produced no remote-point concentration difference resolvable at the resolution of the retained outputs and did not materially alter the approximately 4 h internal-point decay time. The Finer tetrahedral setting also eliminated the coarse-mesh warnings and was therefore used for the reported simulations.

The results support the use of the capsule architecture as a candidate design platform for further drug-specific modeling and experimental validation. They do not demonstrate therapeutic drug-delivery performance, controlled release, biocompatibility, or anticancer efficacy.

## Figures and Tables

**Table 1:** Model parameters and COMSOL Multiphysics 5.2 settings used for the preliminary diffusion model.

Simulation Parameter	Value or Setting
Software	COMSOL Multiphysics 5.2
Physics module	Transport of Diluted Species (tds)
Study type	Time-dependent
*D_eff_* (m^2^/s)	1 × 10^−9^
Porosity *ε*	0.30 (nominal context only; not used to calculate *D_eff_*)
Tortuosity *τ*	1.5 (nominal context only; not used to calculate *D_eff_*)
Nominal peak initial concentration scale, c_0_ (mol/m^3^)	1.0 at the geometric center of the smoothed initial field
Nominal far-field concentration (mol/m^3^)	0.0
Requested output times	0–6 h at 0.1 h output intervals for the main no-flux profiles; additional no-flux reference output at 10 h and open-wall cases extended to 12 h
Mesh type	Free tetrahedral; Finer setting used for reported simulations
Fluid velocity *u* (m/s)	0 (pure diffusion, no advection)
Outer boundary (standard)	No-flux (Neumann): −*n*·*N* = 0
Outer boundary (open-wall case)	Dirichlet condition, c = 0 on selected sink-facing face

**Table 2:** Previously reported geometric and surface parameters of the KTD and KTM capsule variants used as design context for the present FEM study [21]. The reservoir and channel-network descriptors summarize the architecture represented qualitatively in Figure 3; they are not quantitative micro-CT-derived transport descriptors.

Parameter	KTD Capsule	KTM Capsule
Outer dimensions (mm)	6.2 × Ø11.2	4.5 × Ø8
Wall thickness (mm)	1.23	0.85
Surface opening Ø (mm)	0.50	0.40
Spacing between holes (mm)	1.3	1.0
Surface roughness Rz (µm)	118.9	118.9
Internal drug chamber	Central	Central
Channel-network type	Radial + axial	Radial + axial

**Table 3:** Quantitative comparison of representative control-point outputs for the Normal and Finer meshes. Values are reported at the resolution supported by the retained COMSOL 5.2 outputs.

Output Metric	Normal Mesh	Finer Mesh	Comparison
Point 1369, concentration at 6 h (mol/m^3^)	0.002	0.002	No difference resolvable at the retained output resolution (≈0.0002 mol/m^3^)
Point 724, practical near-zero decay time	Approximately 4 h	Approximately 4 h	Same time scale; No material shift

## Data Availability

The original contributions presented in this study are included in the article. Further inquiries can be directed to the corresponding author.
